# Allergic Rhinitis and Chronic Daily Headaches: Is There a Link?

**DOI:** 10.1007/s11910-016-0631-z

**Published:** 2016-02-22

**Authors:** Anna Gryglas

**Affiliations:** Department of Neurology, Gromkovski Voivodship Hospital, Department of Social Pediatrics, Wroclaw Medical University, Wroclaw, Poland

**Keywords:** Allergic rhinitis, Sinus headache, Chronic daily headache, Chronic migraine, Cranial autonomic symptoms

## Abstract

Allergic rhinitis and migraine remain on the list of the most common diseases affecting adults. Migraines and headaches due to allergic rhinitis are easily confused because the symptoms of both conditions often overlap. Both may occur with sinus headache, nasal congestion, and lacrimation and may worsen with weather changes and exposure to allergens. No precise clinical definition exists for what constitutes a sinus headache, which has always been a diagnostic dilemma. Contrary to popular belief, headache is not a typical symptom of rhinitis. Some studies have shown that up to 90 % of sinus headaches are actually migraines. Nevertheless, patients with self-diagnosed sinus headache self-treat or are treated by primary care physicians and/or otolaryngologists with medications for rhinosinusitis, ignoring the neurogenic causes of the symptoms when most of these patients fulfill diagnostic criteria for chronic migraine. Chronic migraine affects 2 % of the general population and has a significant socioeconomic impact on society, incurring health care costs and diminishing quality of life; therefore, the proper diagnosis and treatment of these headache patients should be a priority.

## Introduction

Both allergic rhinitis and chronic daily headache are common health problems worldwide. Many patients present to headache and otolaryngology clinics with self-diagnosed “sinus headache” that ultimately is found to be chronic migraine [1, 2]. No accurate clinical definition of sinus headache exists; this term usually is applied to patients with bilateral headache/pressure affecting the frontomaxillary region and accompanied by autonomic symptoms, all of which also are present in migraine [3]. Contrary to popular belief, sinusitis is not a common cause of this type of headache; it usually is neurogenic in origin [4–6]. Patients with these headaches report that they have received insufficient treatment for years with medications for rhinosinusitis, usually antihistamines or intranasal corticosteroids [7]. Because of an incorrect diagnosis, these patients have undergone unnecessary diagnostic studies and surgical interventions and have not been receiving appropriate, timely treatment [7]. Although in some cases headache may result from sinusitis [8, 9], up to 90 % of purported sinus headaches fulfill the diagnostic criteria for migraine [1, 3, 10–12]. In a study of 2991 patients presenting to primary care physicians with sinus headache, Schreiber et al. [13] showed that 88 % of them were diagnosed with migraine. Sinus headache also may be a result of the comorbidity of both conditions [8, 9], which is not unusual considering the high prevalence of both diseases.

## Chronic Daily Headache and Allergic Rhinitis: Epidemiology and Burden

Chronic daily headache comprises a group of primary and secondary headache disorders in which headache is present on more than 15 days per month for at least 3 months [14, 15]. Approximately, 3 to 5 % of the US population suffers from chronic daily headaches, and the most frequent of which is chronic migraine [14, 16–18]. This debilitating disorder affects around 2 % of the general population and is the leading cause of outpatient and emergency department visits [19–22]. According to the most recent health surveillance studies by the US National Center for Health Statistics, migraine affects roughly one in seven Americans annually, which is comparable to statistics from the previous 8 years [22]. Surprisingly, a recent US study found that the proper diagnosis of chronic migraine is often elusive: only 20 % of patients who fulfill the criteria are diagnosed with the disorder [23]. Several studies have provided evidence that migraine is one of the diseases that mimic allergic rhinitis [11].

Allergic rhinitis is one of the most common chronic diseases among adults in the USA and is the most common chronic disease among children; it is estimated to affect up to 60 million people in the USA alone [24, 25]. It affects around one in six Americans and is responsible for 3.5 million lost work days and 2 million lost school days per year, generating $2 billion to $5 billion in direct health expenditures annually [26–28, 29•]. Allergies and allergic rhinitis are also a huge health problem in Europe. According to the European Academy of Allergy and Clinical Immunology (EAACI), 150 million Europeans, or one in five, have allergies [30, 31]. In the pediatric population, allergy is estimated to occur in one in three children [32]. Based on these statistics, within the next 10 years, 50 % of Europeans will be affected by allergy [33]. Because of the high prevalence and frequent coexistence of allergic rhinitis and chronic migraine—as well as their similar symptoms, which may lead to diagnostic errors—clinicians must be aware of the diagnostic criteria for each condition to make an accurate diagnosis and to apply the appropriate treatment.

## Diagnostic Criteria of Diseases Associated with Sinus Headache: Neurology and Otolaryngology Guidelines

There are two main systems of classification and diagnostic criteria for sinus headache. The vast majority of sinus headache patients fulfill the International Headache Society (IHS) diagnostic criteria for migraine [1, 3, 11, 15], some of them for chronic migraine, the most common form of chronic daily headache. The *International Classification of Headache Disorders*, third edition (ICHD-3), published by the IHS in 2013, defines chronic migraine as headache on 15 or more days per month for more than 3 months. On at least 8 days during the month, the headaches should have migraine features with or without aura and/or should respond to migraine drug treatment; in addition, a secondary cause of chronic headache must have been ruled out [15]. Migraine may be diagnosed after the fifth attack of headache lasting 4 to 72 h (if untreated or unsuccessfully treated) if the headaches have at least two of the following characteristics—a unilateral location, a pulsating quality, moderate or severe pain intensity, and aggravated by or causing avoidance of routine physical activity—plus at least one of the following: nausea and/or vomiting, photophobia, and phonophobia, not better accounted for by another ICHD-3 diagnosis.

The ICHD-3 classification includes a group of secondary headaches, among which is headache related to rhinosinusitis, under the diagnoses “headache attributed to acute rhinosinusitis” and “headache attributed to chronic or recurrent rhinosinusitis,” with the stipulation that accordingly acute, chronic, or recurrent rhinosinusitis is being diagnosed.

In such cases, neurologists will diagnose headache attributed to acute, chronic, or recurrent rhinosinusitis according to the IHS classification, whereas otolaryngologists and allergists will diagnose rhinosinusitis according to the diagnostic criteria of the American Academy of Otolaryngology–Head and Neck Surgery (AAO-HNS) [34]. Rhinitis may be categorized as allergic, nonallergic, or mixed [35•]. Allergic rhinitis is defined as a chronic inflammatory disease of the upper airway caused by an IgE-mediated inflammatory response of the nasal mucous membranes after exposure to inhaled allergens. It is characterized by the symptoms of rhinorrhea (anterior or posterior nasal drainage), nasal congestion, sneezing, and nasal itching (Table 1) [29•], and it often leads to sinus headache [36]. Allergic rhinitis may be classified according to the temporal pattern of exposure to the allergen: for example, seasonal allergic rhinitis, in which the inflammatory response is related to seasonal aeroallergens (e.g., tree, grass, weed pollens), and perennial allergic rhinitis, in which the response is related to year-round environmental aeroallergens (e.g., dust mites, mold, pet danders, or certain occupational allergens). It also may be classified as intermittent allergic rhinitis, in which symptoms persist for more than 4 days per week and more than 4 weeks per year, and persistent allergic rhinitis, in which symptoms last more than 4 days per week and more than 4 weeks per year [37]. In addition, the latest AAO-HNS clinical practice guideline for diagnosing allergic rhinitis defines a third category: episodic allergic rhinitis, in which a patient has an inflammatory response to something not normally encountered in his or her environment (e.g., a cat at a friend’s house) [29•, 38]. Because both the IHS and AAO-HNS classification systems have limitations, creating an interdisciplinary algorithm for sinus headache patients should be a top priority.

**Table 1:** Diagnostic criteria for rhinosinusitis, allergic rhinitis, and migraine

Disease	Classification system	Criteria
Migraine without aura^a^	ICHD-3	A. At least five attacks fulfilling criteria B–D
		B. Headache attacks lasting 4–72 h when untreated or unsuccessfully treated
		C. Headache has at least two of the following characteristics: unilateral location, pulsating quality, moderate or severe pain intensity, aggravation by or causing avoidance of routine physical activity (e.g., walking or climbing stairs)
		D. During headache, at least one of the following: nausea and/or vomiting, photophobia, and phonophobia
		E. Not better accounted for by another ICHD-3 diagnosis
Chronic migraine	ICHD-3	A. Headache, tension-type–like and/or migraine-like, on 15 days/month for at least 3 months, fulfilling criteria B and C
		B. Occurring in a patient who has had at least five attacks fulfilling criteria for migraine without or with aura
		C. On 8 days/month, headache has migraine features and is relieved by a triptan or ergot derivative
		D. Not better accounted for by another ICHD-3 diagnosis
Headache attributed to chronic or recurring rhinosinusitis	ICHD-3	A. Any headache fulfilling criterion C
		B. Clinical, nasal endoscopic, and/or imaging evidence of current or past inflammatory process within the paranasal sinuses
		C. Evidence of causation demonstrated by at least two: 1. Headache has developed in temporal relation to the onset of chronic rhinosinusitis 2. Headache waxes and wanes in parallel with the degree of sinus congestion, drainage, and other symptoms of chronic rhinosinusitis 3. Headache is exacerbated by pressure applied over the paranasal sinuses 4. In the case of unilateral rhinosinusitis, headache is localized ipsilateral to it
		D. Not better accounted for by another ICHD-3 diagnosis
Headache attributed to acute rhinosinusitis	ICHD-3	A. Any headache fulfilling criterion C
		B. Clinical, nasal endoscopic, and/or imaging evidence of acute rhinosinusitis
		C. Evidence of causation demonstrated by at least two: 1. Headache has developed in temporal relation to the onset of rhinosinusitis 2. Either of the following: (a) Headache has significantly worsened in parallel with worsening of the rhinosinusitis (b) Headache has significantly improved or resolved in parallel with improvement in or resolution of the rhinosinusitis 3. Headache is exacerbated by pressure applied over the paranasal sinuses 4. In the case of unilateral rhinosinusitis, headache is localized ipsilateral to it
		D. Not better accounted for by another ICHD-3 diagnosis
Rhinosinusitis	AAO-HNS	Major Facial pain/pressure Nasal obstruction/blockage Nasal discharge/purulence/ Discolored postnasal drainage Hyposmia/anosmia Purulence in nasal cavity on examination Fever (acute rhinosinusitis)^b^
		Minor Headache Fever (all nonacute) Halitosis Fatigue Dental pain Cough Ear pain/pressure/fullness^b^
Allergic rhinitis	AAO-HNS	An IgE-mediated systemic inflammatory disease with symptoms of rhinorrhea (anterior or posterior nasal drainage), nasal congestion, nasal itching, and sneezing in response to an allergen

^a^Migraine with aura is characterized primarily by the transient focal neurologic symptoms that usually precede or sometimes accompany the migraine headache

^b^Two major factors or one major factor and one minor factor are required to diagnose rhinosinusitis

## Why Sinus Headache Is a Diagnostic Challenge: Similarities and Differences

To explain why migraine and allergic rhinitis are confused with each another, we need to consider the common symptoms, pathophysiology, and social beliefs regarding sinus headache [38]. Both disorders may have the same triggers (e.g., weather changes, inhaled irritants) affecting the same parts of the body (e.g., forehead, nose, frontomaxillary area, ethmoid area, space behind and around the eyes) [3]. Migraine and allergic rhinitis also may have similar symptoms, such as nasal congestion, rhinorrhea, pressure/pain behind the eyes, a feeling of fullness in the head, and lacrimation [39]. These cranial autonomic symptoms accompany migraine in a significant percentage of cases, leading to diagnostic errors [40–44]. It is commonly known that these cranial symptoms typically are associated with trigeminal autonomic cephalalgias (TACs) and are critical components of the diagnostic criteria for TACs. Autonomic nervous system dysfunction also is a primary characteristic of migraine [43, 44]. In conclusion, identifying cranial autonomic symptoms in migraine is crucial in the differential diagnosis of TACs and allergic rhinitis [45]. In a study of 177 patients with migraine, Barbanti et al. [42] found that 45.8 % had autonomic symptoms such as rhinorrhea, nasal congestion, lacrimation, conjunctival injection, and eyelid edema during migraines. Similarly, Guven et al. [46] examined 186 patients with episodic migraine and reported that most developed cranial autonomic symptoms with their headaches. They also noted that autonomic symptoms were more common in menstruation-related migraines. Thus, they hypothesized that hormonal factors might play a role in triggering the trigeminal–autonomic reflex pathway [46]. Although the autonomic symptoms in migraine usually are bilateral, in some cases they may be unilateral [42], which must be kept in mind to avoid confusing migraine with TACs. Also, in chronic migraine, cranial autonomic symptoms occur very frequently. Riesco et al. [47] analyzed a group of 100 patients with chronic migraine and found that the most frequent autonomic symptoms were lacrimation (in 49 % of patients), conjunctival injection (in 44 %), eyelid edema (in 39 %), ear fullness (in 30 %), and nasal congestion (in 20 %). However, despite all these data showing that autonomic symptoms frequently are a concomitant clinical feature of migraine, these symptoms are not included in the current diagnostic criteria.

Another common feature of allergic rhinitis and migraine is their seasonal exacerbation in the spring, fall, and summer months as a result of allergic triggers [35•, 39, 48, 49]. A study from South Korea conducted between 2005 and 2013 showed that 13.5 % of migraine patients reported seasonal exacerbations [48]. Moreover, the costs of migraine treatment are higher during allergy season [49], because many people with migraine experience an increase in headache intensity and frequency due to coexisting allergic rhinitis [50]. Furthermore, ocular and nasal symptoms in allergic rhinitis may vary from day to day, depending on the allergen concentration in the atmosphere [51, 52].

To avoid a misdiagnosis, it is important to realize that the pain characteristics of each condition may differ. Patients describe sinus-related pain as dull and pressure-like; usually bilateral; located in the maxillary, glabellar, periorbital, or frontal regions of the skull; and worse in the morning. Those with migraine, on the other hand, usually describe the pain as throbbing or stabbing, mostly unilateral, moderate to severe in intensity, located in the temporal or retro-orbital area, and not worse during a particular time of day [10].

With regard to age at onset, slight differences exist between allergic rhinitis and migraine. Four of five patients with allergic rhinitis develop symptoms before their 20s, with 40 % becoming symptomatic by the age of 6 years [24]. Migraine is a disease affecting mostly women of reproductive age [22], with data from the American Migraine Study II indicating that its peak prevalence occurs between the ages of 25 and 55 years [11]. Furthermore, although migraine headache symptoms include nasal discharge, it is not thick and purulent as is typical in rhinogenic headache. Fever, chills and sweats, itchy nose, and itchy eyes also are typical in rhinitis but not in migraine [53, 54]. Physical examination findings such as dark areas under the eyes, conjunctivitis, and eczema, as well as the presence of another allergic or atopic disease, such as asthma, suggest allergic rhinitis [55]. On the other hand, nausea and vomiting; hypersensitivity to light, sound, and smells; and typical migraine pain lasting 4 to 72 h, occurring at least 8 days per month, and responsive to triptans support the diagnosis of chronic migraine [15].

## Comorbidity and Pathophysiology

In a retrospective study conducted from 2010 to 2012 in 211 patients presenting to otolaryngologists with symptoms of sinus pressure, pain, or headache, nearly half the patients (48.82 %) were diagnosed as having primary headache disorders. In this study, neurologic and rhinologic disease coexisted in around 28 % of the patients [8]. In a questionnaire study among 30,000 Norwegians aged 30 to 44 years, with a 71 % response rate, Aaseth et al. [56] found that those with chronic rhinosinusitis had a ninefold increased risk of chronic headaches compared with the general population. Similarly, Ku et al. [57] reported that migraine was diagnosed much more often in allergic rhinitis patients (occurring in 34 %) than in the control group without allergic rhinitis (occurring in 2 %). In patients with allergic rhinitis, the development and course of migraine may have a significant allergic component [58]. However, in their study of 536 allergic rhinitis patients, Martin et al. [59] found no association between the prevalence of migraine and the degree of allergic sensitization, although they observed a significant age/immunotherapy interaction. In this study, migraine headaches were less prevalent, frequent, and disabling after immunotherapy and in lower degrees of atopy. Similarly, in a prospective study, Theodoropoulos et al. [58] observed that interictal serum C-reactive protein levels declined and the severity of migraines and rhinitis decreased during sublingual immunotherapy. Immunotherapy induces tolerance to specific allergens through two mechanisms of action: by altering cytokine responses of T helper cells and by promoting IgG and IgA, antibodies that block the binding of IgE to mast cells [59–61]. Data from another study revealed a correlation between serum IgE levels and the severity and frequency of migraines: patients with higher levels of IgE had more severe headaches than those with lower levels of IgE [61]. These data support the hypothesis that migraine severity and disability depend on coexisting allergic rhinitis, which might provide an interesting rationale for treatments targeted at reducing migraine intensity and disability among patients with coexisting allergy and migraine; however, further investigation is needed.

The underlying mechanisms for explaining the association between allergic rhinitis and migraine are complex and not well understood. One hypothesis is that a link between autonomic dysfunction and mast cell degranulation or shared genes is responsible for these diseases [35•]. In the pathogenesis of allergic rhinitis, allergens interact with mucosal membranes in the nasal cavity and paranasal sinuses, activating inflammatory cell response [52]. Antigen-presenting cells in the epithelium and lamina propria of the nasal mucosa break the allergens down into small peptides that are presented by MHC class II molecules to T lymphocytes (T cells) [62]. The T cells produce specific cytokines that further differentiate T cell precursors, activating eosinophils and the synthesis and excretion of IgE by B lymphocytes [63]. Two phases of allergic response may be significant. The early phase includes binding of IgE to specific receptors located on the mast cells and basophils, which stimulates these cells to excrete histamine, proteinases, serotonin, leukotrienes, prostaglandins, and other chemicals [59, 64, 65]. This period represents the beginning of the acute stage of allergic rhinitis, with symptoms such as sneezing, runny and itchy nose, swelling, and itchy and watery eyes. Excessive mucus production in the nasal and sinus cavities causes the feeling of sinus pressure and pain [64]. The late phase of allergic reaction occurs 4 to 8 h after exposure to an allergen and is associated with constitutional symptoms such as fatigue, malaise, headache, and irritability [57, 64, 66, 67].

The pathogenesis of migraine is complex and involves recurring activation and sensitization of the trigeminovascular pathway, intracranial arterial dilatation, neurogenic inflammation, and resultant structural and functional changes in genetically predisposed individuals [68]. In migraine pathogenesis, the most affected part of the nervous system is the trigeminovascular system, consisting of nociceptive trigeminal sensory afferents. These neurons originate in the trigeminal ganglion [69] and extend to the dura through three major branches: the ophthalmic (V1), maxillary (V2), and mandibular (V3) [68]. All three divisions supply somatosensory innervation to specific regions of the face, head, and nasal and sinus cavities. Innervation of the anterosuperior part of the nasal cavity is provided by V1, whereas the posteroinferior part is innervated by V2 [70]. The facial parasympathetic efferents innervate the lacrimal glands, nasal and sinus mucosa, and cerebral blood vessels and are responsible for autonomic cranial symptoms, including the conjunctival injection, lacrimation, nasal congestion, and rhinorrhea that accompany migraine attacks [71, 72]. Certainly, it is well established that allergens can activate trigeminal nociceptors by releasing inflammatory chemicals from dural mast cells, triggering a migraine attack [73–75]. Evidence also exists that histamine may induce migraine attacks by increasing the level of nitric oxide (NO) [36], which is believed to promote migraine attacks through its vasodilator effects via H1 and H2 receptors; this is another inflammatory mediator found in both allergic rhinitis and migraine [57, 76, 77]. Inflammation plays an important role in the complex pathogenesis of migraine as well as of allergic rhinitis [58, 78]. In some patients, migraines are triggered by stress or extended physical activity, both of which lead to production of pro-inflammatory cytokines [79]. Obesity, which is associated with a constant state of low-grade systemic inflammation, is believed to be a major risk factor for chronification of episodic migraine and is an exacerbating factor in migraine headaches [80–83]. All these anatomic and physiologic relationships help explain why differentiating allergic rhinitis from migraine is a diagnostic challenge. However, the reason asthma, allergic rhinitis, and other atopic disorders are comorbidities of migraine in a substantial percentage of cases remains unclear and requires further investigation.

## Conclusions

Although migraine and rhinitis are not life-threatening diseases, they both carry a huge socioeconomic burden worldwide, affecting work and daily activities. Both have a high worldwide prevalence, have a high rate of coexistence, and significantly decrease quality of life and productivity by causing fatigue, headache, cognitive impairment, and sleep problems, particularly when they coexist in the same individual [39]. A common theme in studies of migraine and allergic rhinitis is that their similar symptomatology makes them difficult to differentiate. Bearing in mind that these two conditions may coexist in an individual, allergists and otolaryngologists should consider migraine as a diagnosis in their sinus headache patients, whereas neurologists should consider a nasal or sinus pathology as the cause of headaches in their patients. Therefore, physicians must obtain an accurate medical history, focusing their attention on symptoms (i.e., duration, exposures, magnitude of reaction, patterns, and chronicity), triggers, seasonal variations, environmental influences, allergies, past medical history (e.g., trauma, family, and treatment histories), and current treatments. Once a patient receives the correct diagnosis, the physician can focus on minimizing exacerbating factors and offering adequate treatment. In migraine patients with coexisting allergic rhinitis whose migraines are exacerbated by rhinitis, managing the allergies may reduce the frequency of migraines. Determining the appropriate treatment may substantially improve the quality of life of sinus headache patients and reduce the global burden of chronic migraine and allergic rhinitis [83, 84].
